# W-Band Multi-Aspect High Resolution Range Profile Radar Target Classification Using Support Vector Machines

**DOI:** 10.3390/s21072385

**Published:** 2021-03-30

**Authors:** Tomasz Jasinski, Graham Brooker, Irina Antipov

**Affiliations:** 1 Cyber and Electronic Warfare Division, Defence Science and Technology Group, Edinburgh, SA 5111, Australia; irina.antipov@dst.defence.gov.au; 2 Australian Centre for Field Robotics, University of Sydney, Camperdown, NSW 2006, Australia; g.brooker@acfr.edu.au

**Keywords:** millimeter-wave (mmW) imaging, radar target classification, automatic target recognition (ATR), high-resolution range profile (HRRP), support vector machine (SVM)

## Abstract

Millimeter-wave (W-band) radar measurements were taken for two maritime targets instrumented with attitude and heading reference systems (AHRSs) in a littoral environment with the aim of developing a multiaspect classifier. The focus was on resource-limited implementations such as short-range, tactical, unmanned aircraft systems (UASs) and dealing with limited and imbalanced datasets. Radar imaging and preprocessing consisted of recording high-resolution range profiles (HRRPs) and performing range alignment using peak detection and fast Fourier transforms (FFTs). HRRPs were used because of their simplicity, reliability, and speed. The features used were fixed-length, frequency domain range profiles. Two linear support vector machine (SVM)-based classifiers were developed which both yielded excellent results in their general forms and were simple to implement. The first approach utilized the positive predictive value (PPV) and negative predictive value (NPV) statistics of the SVM directly to generate target probabilities and consequently determine the optimal aspect transitions for classification. The second approach used the Kolmogorov–Smirnov test for dimensionality reduction, followed by concatenating feature vectors across several aspects. The latter approach is particularly well-suited to resource-constrained scenarios, potentially allowing for retraining and updating in the field.

## 1. Introduction

It is well-known that radar target imaging is heavily aspect dependent. Consequently, target classification performance can be vastly improved by selecting and combining information from different aspects [1,2]. While multiaspect radar classification have been extensively researched, it is the application of existing multiaspect radar imaging techniques to millimeter-wave radar techniques that has the potential to revolutionize how radar systems are used. Of particular relevance are applications involving multiple unmanned aircraft systems (UASs).

Millimeter-wave radar is well-suited to short-range (i.e., 5–40 km) imaging in clear weather only, primarily due to relatively high atmospheric absorption. Furthermore, it is difficult to achieve high power levels and develop high-power, high-sensitivity receivers and low-loss transmission lines at the frequencies discussed in this paper [3]. However, millimeter-wave radar allows small, cheap and high-resolution (in terms of angle, range and Doppler) radar systems to be developed for niche applications using conventional radar imaging techniques.

Such UAS capabilities have already been successfully demonstrated, particularly in the case of synthetic aperture radar (SAR) imaging [4,5]. Furthermore, research is likely to continue which will ensure further radar implementations across UASs [6]. A closely related area that is also attracting significant interest is millimeter-wave UAS communications, which may eventually result in the combination of radar and communications functions [7,8].

It should be noted that radar equipped UASs are not always the most logical choice for performing radar target classification. Current research trends are focused on satellite-based SAR imaging, where proven machine vision techniques have the potential of being successfully applied [9,10]. However, while target detection has been successfully demonstrated with such techniques, ship classification remains an unsolved problem [9,11]. Despite this, there continues to be numerous advanced techniques applied from convolutional neural networks [12] to various other kernel-based methods [13]. The focus has recently shifted towards improving and simplifying preprocessing and feature attraction approaches. It appears that these steps still cannot be avoided, even though this is one of the key motivations for applying modern machine learning approaches [14,15].

The imaging technique considered throughout this paper is the high-resolution range profile (HRRP), one of the simplest and oldest forms of radar imaging. While range Doppler methods such as inverse synthetic aperture radar (ISAR) are generally considered more effective, they are not suitable for such short-range, time-restricted applications because of:

- time required for collection of samples;
- reliability of image generation depends on ship dynamics;
- computation required for processing two-dimensional data;
- number of transmissions required for generating a single image;
- no benefit in demonstrating multiaspect classification for the purpose of this paper;
- short-range imaging would complement traditional, X-band, long-range ISAR imaging.

In terms of classification using HRRPs, recent progress with neural networks has re-energized research. There have been numerous approaches recently investigated involving deep networks, usually taking the form of deep belief networks or stacked denoising autoencoders [16]. These approaches promise to achieve the same levels of effectiveness as traditional approaches requiring extensive human expertise and input, while remaining largely data-driven [17,18]. Other promising approaches include recurrent neural networks [19] and recurrent belief networks [20] which can characterize temporal variations in HRRPs. Some of these approaches claim to operate well at low signal-to-noise ratios (SNRs) [21] and claim to outperform traditional approaches such as principal component analysis (PCA), linear discriminant analysis (LDA) and support vector machines (SVMs) [22]. Other approaches involving autoencoders claim to reduce the need for large quantities of training data which is a traditional problem with neural networks [23,24,25].

In any UAS radar implementation, multiaspect radar classification is bound to play an important role. While much previous research has been carried out [1,2,17,26,27,28], its application to dynamic, short-range scenarios where the radar can control which aspects are being imaged has not been made. The majority of previous work has focused on long-range scenarios where the orientation of the target cannot be effectively controlled, and the radar can only gather data from a few contiguous aspects that are presented to it.

An initial intuitive approach for performing multiaspect radar classification would be to treat each aspect as an independent classification, combining classifier results via weighting or averaging. However, such an independent approach does not yield results as effective as methods that treat different aspects as dependent [2]. This is intuitive since the radar measurement from any aspect is a function of the structure of the target which is common to all aspects, even though the visible scatterers may differ because of occlusion. A different class of methods are required to make the most of dependencies between aspects.

A common approach for performing multiaspect radar classification is the hidden Markov model (HMM) [1,26,27,28,29]. This approach offers many advantages, such as its prevalence and simplicity in implementation. A hidden Markov mode is characterized by an internal unknown (hidden) state, which in this case most logically corresponds to the target aspect. The observations are the radar measurements which give a probabilistic indication of what the state is. It is also characterized by being dependent only on the observation and the previous belief or message. No history needs to be recorded, allowing it to be efficiently applied.

The major drawback of the HMM approach is that there is typically a reduction in feature fidelity caused by the need to maintain its computational simplicity. There are many techniques commonly used for representing signals by a finite set of basis functions or kernels, such as matching pursuits and the relaxation algorithm [1]. Furthermore, features are often quantized into discrete symbols. Both actions can hamper the fidelity of the classification system [30].

Another intuitive and relevant approach for performing multiaspect target classification is the feature space trajectory concept [2,31]. This approach generally relies on applying feature extraction before normalizing features onto a feature space that can be analyzed using geometric means. This is a flexible and intuitive approach allowing for customization and optimization. However, the key question that remains unsolved is how effective it is compared to other methods, particularly in complex scenarios.

This paper takes a closely related and well-established approach: the SVM [32,33]. While the SVM is also intuitive, it is a well-established approach that utilizes kernels designed for each application and although this approach suffers from the disadvantage of training complexity which can involve engineering a kernel function, once trained the SVM offers fast classification through several basic operations. While this approach is not the latest being researched [16,17,18,19,20,21,23,24,25], it has several advantages relating to this particular application, particularly when compared to deep learning methods [34]:

- it is far easier to determine parameters in an SVM;
- training time is significantly shorter for the SVM;
- less training data are required for the SVM;
- depending on the application, SVMs often result in better accuracy;
- SVMs are better suited to active learning schemes, where data for training is specially selected for training.

Furthermore, there are numerous cases where SVMs have outperformed neural network approaches at tasks of similar complexity such as [35,36,37]. Therefore, given the current state of research, the SVM is probably a more robust and appropriate solution for this application. However, this may not be the case soon as deep learning approaches quickly evolve and mature.

The work presented here has been preceded by [38], where simulated data were used. Four time domain classifiers were investigated (listed from most effective to least effective): correlation, radial basis function (RBF) kernel SVM, polynomial kernel SVM, and naïve Bayes. The correlation classifier was tailored for a time domain simulation where range alignment was not a concern. The approach is not suitable for real data unless range alignment can be adequately addressed. The two SVM approaches have been tested in this paper along with a linear kernel SVM, which is the focus. The naïve Bayes approach was the poorest in terms of performance and consequently has not been considered in this paper.

The aims of this paper are therefore to develop a robust, efficient and fast, multiaspect classifier extension to previous SVM-based work and demonstrate the technique on real radar data. The work builds on previous single and multiaspect HRRP radar classification research and tailors it for future UAS applications that will be characterized by short ranges, dynamic flight trajectories, limited resources, limited data and distributed sensors. These applications will be complicated by the requirement to provide covert radar operation and covert UAS-to-UAS communication links. Such scenarios will demand that minimal transmissions are made, information collected from each aspect can be readily fused and classifiers can be retrained in the field.

Three approaches utilizing the linear SVM classifier are presented in this paper. The first approach demonstrates the effectiveness of the linear SVM classifier in a single classification against the entire dataset. The second approach demonstrates the performance improvement that can be expected by splitting data into sectors and prioritizing aspects. The third approach demonstrates that combining feature spaces across aspects can efficiently achieve excellent results from only a few features while inherently allowing for small and imbalanced datasets.

Details of the radar data collection are presented in Section 2. A description of the feature extraction process is presented in Section 3. The developed classifiers are presented in Section 4.

## 2. Radar Measurements

Radar measurements were conducted using a W-band radar system configured to operate as a frequency modulated continuous-wave (FMCW) radar. The radar system was mounted on the balcony of a control tower of a major port at a height of about 15 m above sea level. The typical range to target was 600 m, giving a slight depression angle. The collected data therefore correspond to a single elevation angle only.

Two boats were fitted with attitude heading and reference systems (AHRSs) and instructed to perform slow turns so that radar data could be collected for 360°. The AHRS data, which consisted of yaw/heading, pitch, roll and position, were time-stamped with GPS and recorded to a memory card. The radar system was connected to a GPS time server and radar data were also time-stamped to GPS time. 

The radar was configured to transmit frequency chirps over a 400 MHz bandwidth, giving a theoretical range resolution of 37.5 cm. High-resolution range profiles (HRRPs) were logged over the duration of the experiment. Further technical details concerning the AHRS and radar data collections are shown in Table 1. Each radar burst consisted of 16 chirps, which were transmitted end-to-end. The profile from the first chirp of each frame was logged, as well as the average profile from all 16 chirps in the frame. Throughout this paper, only the average collection has been used.

In order to reduce data processing requirements and ensure the fastest possible update rate, a stretch processing IF receiver was employed for this trial. This allowed a sampling rate of 1 MHz to be employed (rather than 400 MHz using a conventional in-phase quadrature (IQ) receiver), resulting in an unambiguous range of 3750 m.

**Figure 1:**
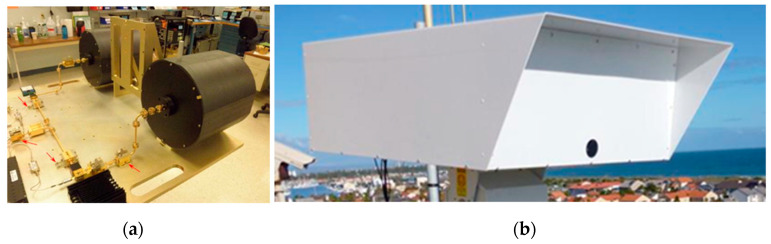
(**a**) The internal design of the W-band radar showing transmit and receive antennas and RF section; (**b**) the deployed radar encapsulated by a radome mounted on a pan-tilt positioner and situated at the top of a control tower at the entrance to a busy port.

Tracking of the targets was achieved by the operator using a joystick to control the antenna position. A bore-sighted video feed allowed this to be carried out precisely. The internal layout of the RF front-end and the external mounting are shown in Figure 1. 

**Figure 2:**
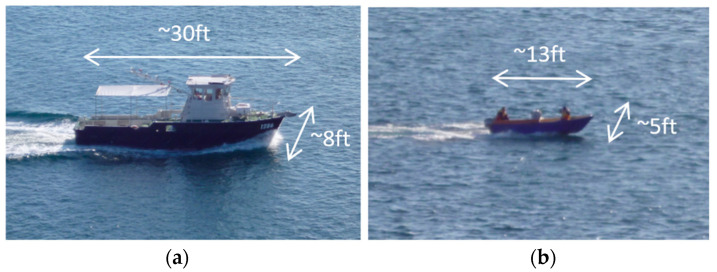
(**a**) The “Investigator” test target; (**b**) the “Tender” test target.

Two targets were utilized for the experiment: the higher radar cross-section (RCS) “Investigator”, which was a dive boat with an enclosed cabin approximately 30 ft in length, and the lower RCS “Tender”, which was an open dinghy approximately 13 ft in length. Both targets are shown in Figure 2. Approximately two full revolutions of measurements for each boat were made, lasting approximately 13 min for each target.

**Figure 3:**
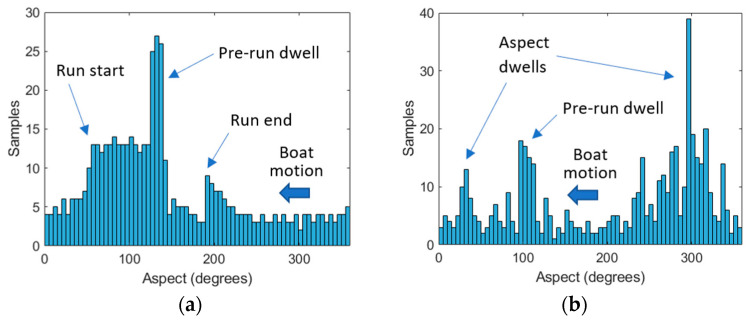
Histograms showing range profiles collected as a function of aspect angle, in five-degree bins for (**a**) the Investigator test target; (**b**) the Tender test target.

A total of 516 range profiles were collected for Investigator and 525 range profiles for Tender. Owing to the manual control of the boats and inconsistent rates of rotation, as well as dwell times at the beginning and at the end of the run, the data were not collected evenly over all aspects. Figure 3 shows the distribution of profiles collected for each target in 5° bins as a function of aspect. At some aspects, as many as 25 profiles were collected for Investigator and 35 for Tender. At other aspects, less than five were available for both targets. This implies that datasets for classification will be highly imbalanced.

## 3. Feature Extraction

The feature extraction phase aims to prepare the collected raw data for classification. The general approach throughout the feature extraction stage was to avoid quantization and parameterization of data as much as possible, allowing subtleties in data to remain. 

For the reasons described above, the radar imaging technique used in this paper is the HRRP. One of the problems when using this radar imaging technique for classification is range alignment. To combat this problem, all subsequent processing was carried out in the frequency domain, which removes any small variations in time domain misalignment, as explained and implemented by Jasinski et al. [38] and Zyweck et al. [39].

**Figure 4:**
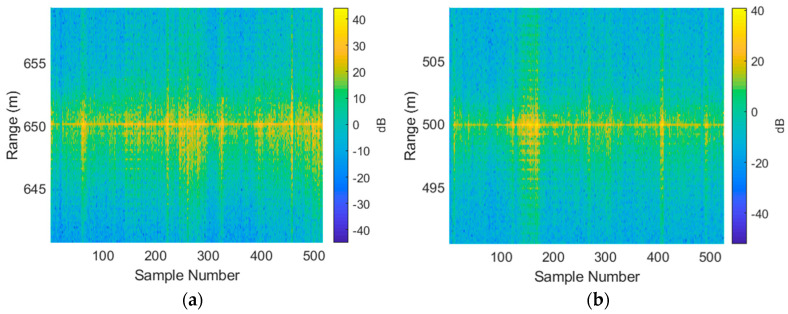
All collected data shown in terms of range-aligned range profiles for (**a**) the Investigator test target; (**b**) the Tender test target.

**Figure 5:**
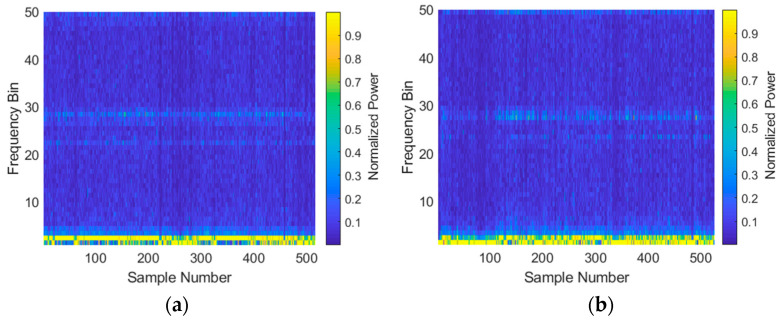
All collected data shown in terms of frequency domain profiles for (**a**) the Investigator test target; (**b**) the Tender test target.

The following steps were carried out to convert raw AHRS and radar data into feature vectors that could be utilized by the classifier:

1. Alignment of AHRS and radar data according to GPS time: since the AHRS data were collected more frequently, the average of all recorded headings throughout the radar measurement was used.
2. Peak detection: this was used to align the profiles in range. One risk with this step was that speckle noise from outside the area of interest would corrupt the peak detection, but this did not occur. Another disadvantage was that different parts of the structure could become dominant throughout target rotation, making the target track in range stepped. However, given the frequency domain processing applied here, this is of no consequence.
3. Cropping all data to a region of interest: in this case, data were cropped to achieve a 50 range-bin span which corresponds to 50 × 0.375 = 18.75 m. The results from this step are shown in Figure 4.
4. Taking FFTs (absolute value only and discarding half of the resulting data) and normalizing range profiles to obtain feature vectors. The result of this step is shown in Figure 5.

The resulting feature vectors were simply the frequency domain range profiles of length 50. For each radar sample (i.e., transmission), one range profile was obtained and one feature vector was produced. Experiments using the same radar system but different targets in [33] compared linear SVM, LDA and correlation classifiers in the time and frequency domains. The study showed that for real radar data, the SVM classifier in the frequency domain worked the best.

Two further variables that became apparent while conducting the preprocessing were whether to normalize the frequency domain data and whether the feature vectors should be represented in log or linear domains ahead of the classification. The four possibilities each resulted in slightly different classification results. This investigation is summarized in the subsequent section on classification.

**Figure 6:**
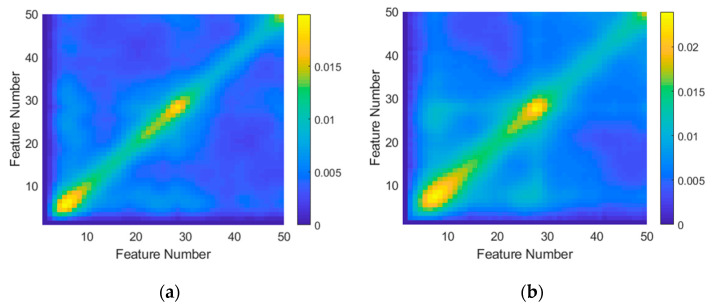
Covariance matrices generated for (**a**) the Investigator test target and (**b**) the Tender test target using frequency domain data. For this calculation, linear preprocessing with normalization was utilized.

Although not strictly part of the feature extraction, as a final inspection of the preprocessed data, covariance matrices were generated for both targets. These are shown in Figure 6. The X and Y dimensions correspond to the extracted feature vectors with lengths of 50. Each data point represents variance calculated between the X and Y feature numbers for the full dataset. The diagonal represents covariance across the entire feature vector. The peaks at the beginning and end represent the DC offset to the profiles (due to RCS fluctuation). The peak at the center corresponds to a frequency component at half of the sampling frequency, possibly resulting from nonlinearities in the radar system. This depiction can provide insight into features covarying with other features for each target. Of particular interest are the regions outside of the diagonal where there are numerous smaller peaks. These peaks can potentially be used for dimensionality reduction since they identify those features that covary.

## 4. Classification

As a first step, an estimate of signal-to-noise ratio was made to assess likely classification performance. This was carried out by averaging all power in the region of interest and comparing to a test area which did not contain target energy. The average signal-to-noise ratios were 29 and 23 dB for Investigator and for Tender, respectively, which was at least 10 dB higher than the signal-to-noise ratio of 12 dB considered as part of the simulations in [38], demonstrating an average SVM accuracy of about 0.6. At SNRs of 23–29 dB, an SVM accuracy exceeding 0.97 was achieved for simulated data.

While the SVM classifier is the focus of this paper, as a starting point, a simple hard-threshold power level classifier was implemented as a useful reference. Since the targets were of substantially different RCSs, a reasonable level of classification performance can be expected simply by making a measurement of power level.

A Markov process classifier was also implemented as a reference, but this performed poorly. No further discussion of this classifier is provided in this paper.

The SVM classification progression shown in this paper involved starting with simple techniques acting on the full dataset and gradually extended to more complicated techniques that divide the data into small subsets, eventually performing multiaspect classification and combination of feature spaces.

### 4.1. Hard Threshold Power Classifier

**Figure 7:**
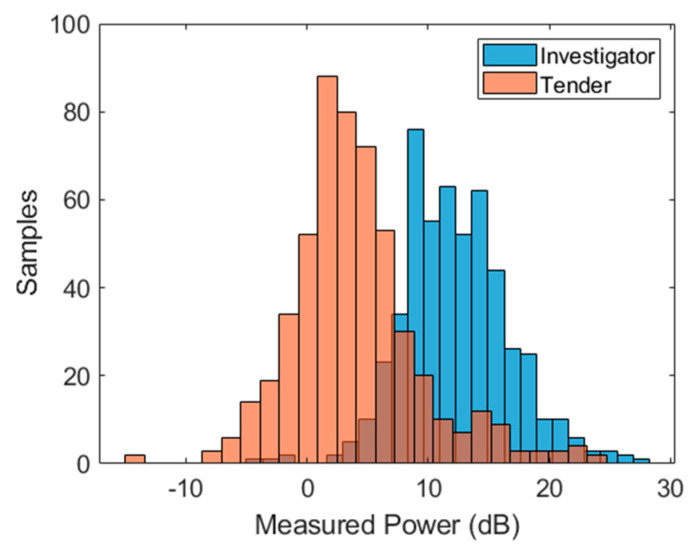
Histogram of average power in measurement window for all collected data for both targets.

Given that the two target types involved a lower-RCS and higher-RCS target, it is intuitive to perform a simple hard threshold classification using the mean power level alone. This will prove to be a useful reference in terms of classifier performance. Subsequent, more advanced classification techniques utilizing features vectors should be able to achieve a significantly better classification result. A histogram of the total power in the time domain for both targets is shown in Figure 7. The average power received for the Investigator target was approximately 12.5 dB. The average power for Tender was approximately 2.5 dB. It should be noted that the average range-to-target for both sets of measurements differed (approximately 650 m average range for Investigator and approximately 500 m average range for Tender). Assuming an R^4^ range-to-RCS dependence, this difference would have separated the two histograms by 4.6 dB (with Tender above Investigator), if the RCSs of the two targets were the same.

A hard threshold of 7.7 dB was used to perform a power level classification. The confusion matrix and key performance derivations are shown in Table 2.

The performance derivations used above and throughout this paper include the following:

(1) $$\mathrm{Accuracy}=\frac{\mathrm{TP}+\mathrm{TN}}{\mathrm{TP}+\mathrm{FP}+\mathrm{TN}+\mathrm{FN}}$$

(2) $$\mathrm{True}\;\mathrm{Positive}\;\mathrm{Rate}\;\left(\mathrm{TPR}\right)=\frac{\mathrm{TP}}{\mathrm{TP}+\mathrm{FN}}$$

(3) $$\mathrm{True}\;\mathrm{Negative}\;\mathrm{Rate}\;\left(\mathrm{TNR}\right)=\frac{\mathrm{TN}}{\mathrm{TN}+\mathrm{FP}}$$

(4) Informedness=TPR+TNR−1

where TP, FP, TN and FN are the true positive, false positive, true negative and false negative statistics, respectively. Accuracy is used as a general performance measure, indicating the ratio of correct classifications to all classifications. The true positive rate (TPR) indicates the ratio of correctly classified Investigator samples to all Investigator samples, while the true negative rate (TNR) indicates the ratio of correctly classified Tender samples to all Tender samples. Informedness gives the probability of the classifier making an informed decision. An informedness of zero is representative of random guessing, while a positive informedness indicates that the performance of the classifier is informative.

### 4.2. Full Dataset SVM Classifier

Prior to dividing the data into subsets, a classification of the entire dataset was performed using a linear SVM classifier. The SVM uses a hyperplane to hard classify test points belonging to two classes. The separator can be written as [40]:

(5) w·x+b=0

where **w** is a weight vector, **x** is the vector of features, and *b* is an intercept scalar offset. Finding the optimal hyperplane is achieved by solving the equation:

(6) $$\underset{\alpha }{\arg\max}\underset{j}{\sum }\alpha_{j}-\frac{1}{2}\underset{j,k}{\sum }\alpha_{j}\alpha_{k}y_{j}y_{k}\left(x_{j}\cdot x_{k}\right)$$

which is subject to the constraints $\alpha_{j}\geq 0$ and $\sum_{j}\alpha_{j}y_{j}=0$, where *α* is a weight vector and *y* is the class vector. The decision function can then be written:

(7) $$h\left(x\right)=sign\left(\underset{j}{\sum }\alpha_{j}y_{j}\left(x\cdot x_{k}\right)-b\right)$$

A total of 990 profiles were used for training and 52 for testing which represents a 5% holdout. This classification was carried out for the four configurations discussed above (log with normalization, log without normalization, linear with normalization and linear without normalization). The classification results are shown in Table 3, Table 4, Table 5 and Table 6, respectively.

In terms of accuracy, all combinations performed similarly with the exception of linear with normalization, which performed relatively poorly. One of the better performing configurations was log classification with normalization. In addition to its strong performance, the datasets were all in the 0–1 range, which made them easier to work with. This is the configuration that has been used for the remainder of the paper.

As a final assessment of the effectiveness of the linear kernel SVM, the same dataset was applied to both an RBF kernel SVM and a polynomial kernel SVM, previously applied in [38]. The results from these classifiers were comparable, with the best performing kernel being a function of which data were being held out. Averaging over 30 random holdout iterations, an accuracy of 0.86 and 0.84 was achieved for the RBF and polynomial kernels, respectively, compared to 0.87 for the linear classifier.

### 4.3. Multiaspect SVM

The first multiaspect classification attempt was the most intuitive one, dividing the full dataset into sectors and performing classification on each sector independently. This approach can be considered as independent since the outcome at each sector is independent of any other sector.

When conducting the multiaspect classification, it was apparent that the 1041 range profiles collected in total were insufficient for a high-fidelity classification with the small intervals (e.g., 5°) typically used in such applications. For this reason, the sector width was increased to 30° so that each classifier had adequate data for thorough training and testing. The trade off in this approach was that each dataset consisted of data with more dramatic fluctuations, making the classification more challenging for the classifier. Furthermore, the holdout was increased from 5% to 25% so that adequate data were available for testing. 

Throughout the multiaspect testing, two further performance derivations were introduced:

(8) $$\mathrm{Positive}\;\mathrm{Predictive}\;\mathrm{Value}\;\left(\mathrm{PPV}\right)=\frac{\mathrm{TP}}{\mathrm{TP}+\mathrm{FP}}$$

(9) $$\mathrm{Negative}\;\mathrm{Predictive}\;\mathrm{Value}\;\left(\mathrm{NPV}\right)=\frac{\mathrm{TN}}{\mathrm{TN}+\mathrm{FN}}$$

The positive predictive value (PPV) gives the ratio of correctly classified Investigator samples to all Investigator classifier outcomes, while the negative predictive value (NPV) gives the ratio of correctly classified Tender samples to all Tender classifier outcomes. These metrics will be important further in this paper when considering transition probabilities for both target types.

**Figure 8:**
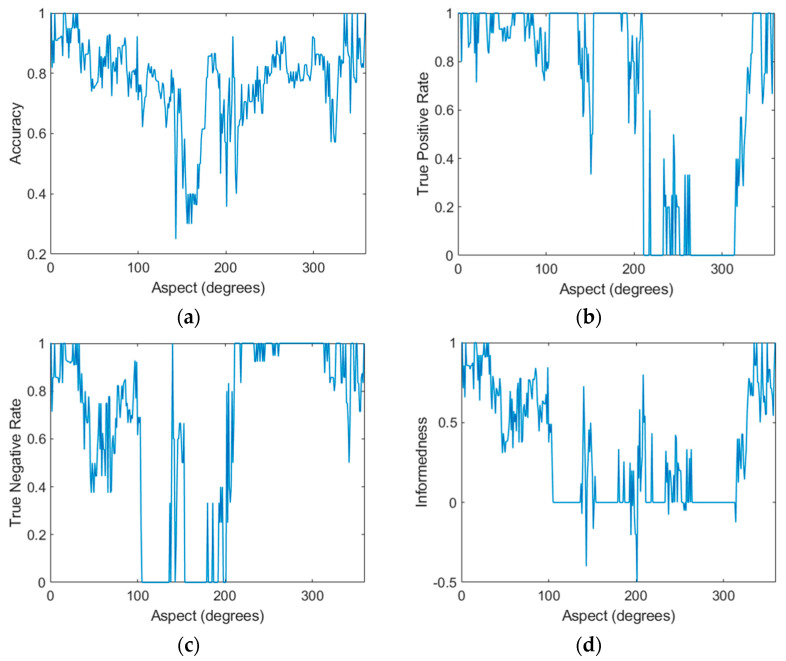

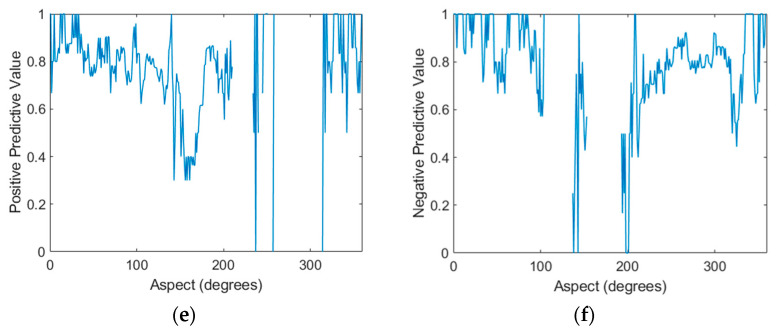
Key performance derivations for the multiaspect linear support vector machine (SVM) classifier using a 30-degree classification window in one-degree steps and 0.25 holdout including (**a**) accuracy, (**b**) true positive rate (TPR), (**c**) true negative rate (TNR), (**d**) informedness, (**e**) positive predictive value (PPV), and (**f**) negative predictive value (NPV).

Although a sector width of 30° has been used in the multiaspect classification, the SVM classification was carried out at 1° intervals with a moving 30° window. For each step, a random selection of training and test points were made. Figure 8 shows all six performance derivations at each aspect. An aspect of zero degrees indicates the bow (nose) of the target. 

In terms of accuracy, 100% accuracy was achieved within the 335–35° range. Good accuracy was also achieved in the 60–105° and 220–305° ranges. Accuracy was generally highest when the target was imaged along its length.

There were aspects at which the TPR and TNR fell to zero (and rose to one for the other target), indicating that the classifier always selected one target type. This clearly represents aspects that fail to achieve any level of worthwhile classification. This is best illustrated in the informedness plot by the regions where informedness falls to zero (i.e., the classifier does not provide any information) or lower. These problematic aspects were: 100–130°, 155–190°, 210–230°, and 260–305°. Generally, these sectors represent aspects where the target was broadside or stern (rear) to the radar. Conversely, and consistent with the accuracy plot, it was the aspects from 0–100° and 310–0° that resulted in high informedness. This finding confirms that multiaspect imaging is highly dependent on aspect and identifies a particular set of aspects that are worthwhile for classification. A total of 54% of aspects (those aspects where informedness was positive) were effective for target classification given this particular dataset, preprocessing scheme and classifier. 

While there was some symmetry about zero degrees, the plots were not entirely symmetrical, indicating different performance depending on which side of the target was being imaged. This is a common characteristic of millimeter-wave radar measurements where the RCS is very sensitive to small structural features, particularly flat plates, dihedral and trihedral reflectors [41].

#### Combining Observations

In order to apply the SVM classifier developed in the previous section to dynamic multiaspect scenarios, target observation probabilities, *P*(*X*|*O*), for each target at each target aspect are required. These are required not only to determine the final, multiaspect target probability (using the sum or product rules described later in this section) but also to provide a mechanism for prioritizing aspects where the classifier is most likely to achieve correct classification.

Unfortunately, the SVM classifier does not inherently provide an output in terms of a probability. The distance to the SVM hyperplane in feature space is generally not regarded as a reliable measure. One common solution is fitting a logistic function using logistic regression to the training sets and then applying the function to each test point. This approach relies on parameterization of the dataset which may not be effective for such a small amount of data. Ideally, a nonparametric approach is sought here.

We propose using the training set statistics directly—specifically, the true positive rate (TPR) and true negative rate (TNR) defined in Equations (2) and (3) to generate observation probabilities, and the positive predictive value (PPV) and negative predictive value (NPV) defined in Equations (8) and (9) to generate posterior (target) probabilities.

For the Investigator target, the observation probabilities are therefore:

(10) $$P\left(O_{n}=I|X_{n}=I\right)=\frac{{\mathrm{TP}}_{n}}{{\mathrm{TP}}_{n}+{\mathrm{FN}}_{n}}={\mathrm{TPR}}_{n}$$

and for the Tender target, the observation probabilities are:

(11) $$P\left(O_{n}=T|X_{n}=T\right)=\frac{{\mathrm{TN}}_{n}}{{\mathrm{TN}}_{n}+{\mathrm{FP}}_{n}}={\mathrm{TNR}}_{n}$$

where *n* is the target aspect or state, and TP, FP, TN and FN are the true positive, false positive, true negative and false negative statistics for the corresponding target and target state or aspect.

Intuitively (or using Bayes rule), the posterior (target) probabilities for Investigator are:

(12) $$P\left(X_{n}=I|O_{n}=I\right)=\frac{{\mathrm{TP}}_{n}}{{\mathrm{TP}}_{n}+{\mathrm{FP}}_{n}}={\mathrm{PPV}}_{n}$$

and for Tender target, the posterior (target) probabilities are:

(13) $$P\left(X_{n}=T|O_{n}=T\right)=\frac{{\mathrm{TN}}_{n}}{{\mathrm{TN}}_{n}+{\mathrm{FN}}_{n}}={\mathrm{NPV}}_{n}$$

**Figure 9:**
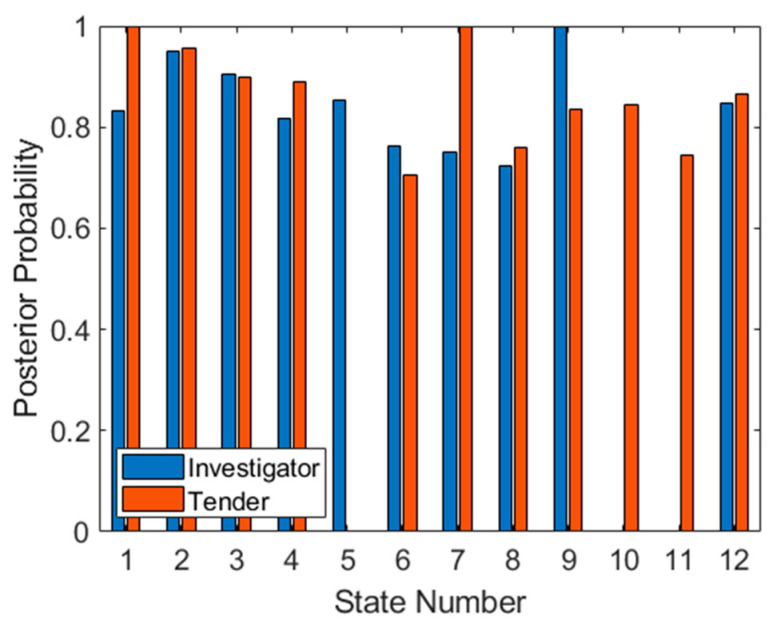
Posterior probabilities for both targets at each aspect for the training set.

This approach assumes that the linear SVM will misclassify not only test points, but also training points, implying that it may be more effective using a “weak” classifier when implementing multiaspect classification. A linear SVM in high-dimensional hyperspace may fit this requirement well. Figure 9 shows the posterior probabilities for each of the targets at each aspect, using the training set to obtain PPV and NPV statistics. These posterior probabilities indicate the probability that the classifier will correctly classify each target at each aspect (i.e., the ideal plot would have all values at unity, with no misclassifications).

While some aspects are not appropriate for classification (such as 5, 10 and 11) the other aspects (such as 1, 2, 3, 4, 6, 7, 9 and 12) promise reliable classification as well as high target probabilities.

**Figure 10:**
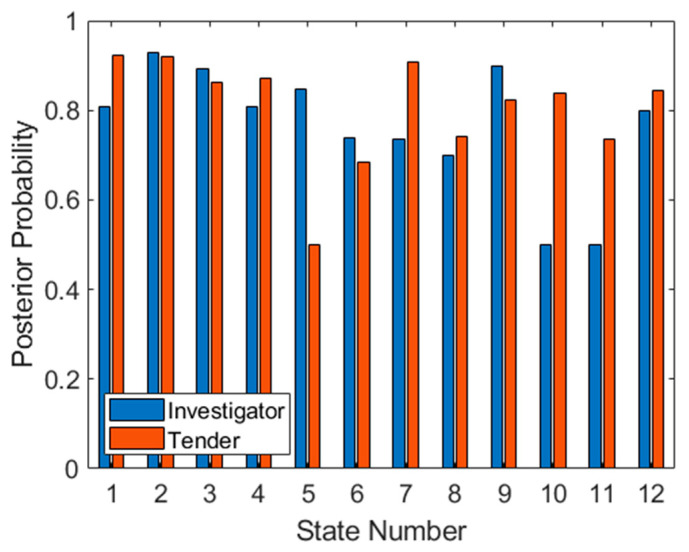
Smoothed posterior probabilities for both targets at each aspect for the training set.

One problem evident in the posterior probabilities in Figure 9 is that there are probabilities at zero and one. Both numbers are unrealistic and will cause problems when later using the product or sum rules to accumulate probabilities. The solution employed here, which is common to this type of problem, is applying additive (or Laplace) smoothing to the data. The general form of this method is:

(14) $${\hat{\theta }}_{i}=\frac{x_{i}+\alpha }{N+\alpha d}$$

where ${\hat{\theta }}_{i}$ is the smoothed probability for class *i*; *x_i_* is the data to be smoothed (in this case PPV × (TP + FP) for Investigator and NPV × (TN + FN) for Tender, both corresponding to training data only); *N* is the number of samples (in this case TP + FP for Investigator and TN + FN for Tender, again only corresponding to training data); *α* is the smoothing parameter (in this case one) and *d* is the total number of classes (in this case two). The result of this operation on the training data in Figure 9 is shown in Figure 10. Unusually, the zero probabilities of states 5, 10 and 11 have been now raised to 0.5. However, on further inspection, this is caused by no negative (i.e., Tender) classifications in the training set for state 5, and no positive (i.e., Investigator) classifications on states 10 and 11, so NPV and PPV statistics cannot be generated and the probability can only be a random guess (or the prior probability).

Once the posterior probabilities have been obtained for both targets at all aspects, it is possible to prioritize aspects based on expected classifier performance. Although there are several metrics for achieving this, in this case we have simply multiplied the smoothed observation probabilities together at each aspect, and sorted from the highest product to the lowest. This results in an optimal aspect sequence of 2, 3, 1, 9, 4, 12, 7, 8, and 6. The final three aspects (5, 10 and 11) have been omitted since one class at each of these aspects has a posterior probability of 0.5, and therefore these aspects are not any better than random guessing (for at least one target). Once again, this metric is somewhat arbitrary and depends on the application. In fact, one of the benefits of this approach which has not been exploited here, is that probabilities from different aspects can be combined, through selection of aspects, to obtain the desired receiver operating characteristic (ROC) statistics, depending on the objective of the classification (for example, minimizing false positives from a particular target).

**Figure 11:**
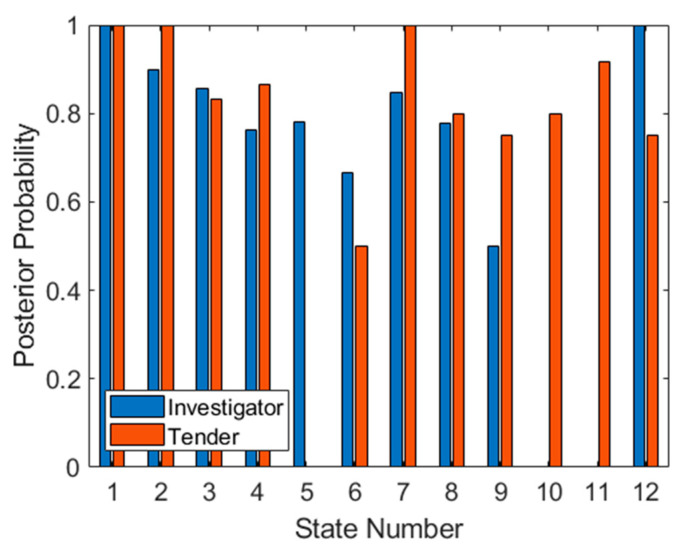
Posterior probabilities for both targets at each aspect for the test set.

The test set has been applied to the trained SVM in Figure 11, firstly as a method of confirming that the observation probabilities are reliable. The similarity of both plots (the training set in Figure 9 and the test set in Figure 11) suggests that the datasets are indeed identically distributed. It is also possible to use the test set to predict how well the derived sequence will work. As seen in Figure 9, aspects 5, 10 and 11 are ineffective for classification since one target type cannot be classified. If we apply the previously determined optimal sequence to the data in Figure 11, we see that for the first three aspects (2, 3 and 1), classification of both targets is correct (assuming that all available test data are considered). However, at aspect 9, a posterior probability of 0.5 is achieved for Investigator. For the next four aspects (4, 12, 7 and 8) classification performance is well above 0.5 for both targets. Finally, for the last prioritized aspect (6), the posterior also falls to 0.5 for the Tender target, making this aspect ineffective.

We can therefore conclude that when using this technique, aspects should be prioritized using the products of their posterior probabilities. To achieve a monotonically increasing target probability (assuming use of the product rule to combine posterior probabilities across aspects, discussed later in this section), the best three (out of twelve) aspects should be used. Otherwise using all nine valid prioritized aspects will also provide further gains but the process will involve more measurements from more aspects. However, in this case because of 0.5 posteriors at two aspects, this accumulated target probability will not improve when transiting these aspects, so confidence in target class will increase more slowly.

We thus obtained the necessary posterior (target) probabilities for both targets across all states, providing us with an optimized sequence of aspects and a valid test set. The problem now becomes combining classification results to achieve a confidence measure.

There are many options for combining classification results. Two common approaches are the sum and product rules. The sum rule is better suited to dependent feature spaces, with potentially large errors. The product rule is preferable when classifiers have small errors and are operating in independent feature spaces [42]. In this paper, we will therefore use the sum rule for combining probabilities within a single aspect (where observations are likely to be dependent and fluctuations may be high) and we will use the product rule to combine probabilities between aspects (where measurements may be regarded as somewhat independent and errors are small).

We define the sum rule in this context as:

(15) $$P\left(X|O_{p}\right)=\frac{1}{N}\overset{N}{\underset{n=1}{\sum }}P\left(X|O_{n}\right)=\frac{1}{N}\overset{N}{\underset{n=1}{\sum }}{\hat{\theta }}_{i}\left\{h\left(n\right)\right\}$$

where *P*(*X*|***O_p_***) is the combined posterior (target) probability for state *p*, and *P*(*X*|*O_n_*) are the target posterior probabilities from Figure 10, and ${\hat{\theta }}_{i}\left\{h\left(n\right)\right\}$ are the set of smoothed target posterior probabilities indexed by the classifier output, corresponding to class *i* for *N* available test points. This calculation was repeated for both targets and all aspects.

We defined the product rule in this context as:

(16) $$P\left(X|O\right)=\frac{\prod_{p=1}^{P}P\left(X|O_{p}\right)}{\sum_{x=1,2}\prod_{p=1}^{P}P\left(X|O_{p}\right)}=\frac{\prod_{p=1}^{P}\sum_{n=1}^{N}{\hat{\theta }}_{i}\left\{h\left(n\right)\right\}}{\sum_{x=1,2}\prod_{p=1}^{P}\sum_{n=1}^{N}{\hat{\theta }}_{i}\left\{h\left(n\right)\right\}}$$

where *P*(*X*|***O***) is the multiaspect, combined posterior (target) probability for *P* states or aspects, normalized for targets *x* = 1,2. *P*(*X*|***O_p_***) is the single-aspect target posterior probability obtained by the sum rule. This calculation is repeated for both targets, *x* = 1,2.

**Figure 12:**
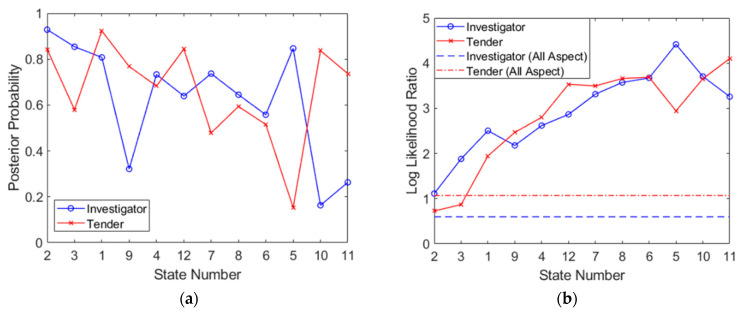
Posterior probabilities plotted for the optimized aspect sequence showing (**a**) results of the sum rule for each aspect, *P*(*X*|***O_p_***), and (**b**) results of the product rule, expressed as a log-likelihood ratio (LLR).

The posterior probabilities obtained from the product rule, *P*(*X*|***O***), are cumulative and with each subsequent aspect can become extremely large. For the purpose of visualization, these are expressed as log-likelihoods. Furthermore, in order to avoid the normalization step, they are expressed as likelihood ratios (the ratio of the likelihood of the correct target to the likelihood of the incorrect target). The log-likelihood ratio can be written as:

(17) LLR=log(P(X|O))−log(P(¬X|O))

where *X* represents the correct class and *¬X* represents the incorrect class (when referring to confusion matrices, this is calculated as LLR_I_ = log(PPV) − log(FOR) for Investigator and LLR_T_ = log(NPV) − log(FDR) for Tender, where FOR is the false emission rate, FOR = FN/(FN + TN) and FDR is the false discovery rate, FDR = FP/(FP + TP)), calculated for both targets. The results of both the sum rule (combining results within a single aspect) and the product rule (combining results from multiple aspects) expressed as a log-likelihood ratio, are shown in Figure 12. These plots have been generated according to the optimized aspect sampling order, as previously detailed. The gradual decrease in target probabilities in Figure 12a confirms that the highest value aspects are indeed sampled first. It should be noted that Figure 12a differs from Figure 11 since it does not utilize test set statistics; these calculations rely on training set statistics, indexed by test set results as they are obtained (as would be the case in a real classification). All available test points at each aspect have been utilized (and the final three poorly performing aspects have also been included for completeness).

Figure 12b shows the progression of the log-likelihood (which can be seen as a confidence measure of each class) through measurements at successive aspects. In order to compare performance to traditional, full dataset classification, log-likelihoods have also been generated from data in Table 3 (full dataset, all-aspect SVM classification) as a reference. The log-likelihood exceeds that of the full dataset classifier for both targets at the third aspect and beyond. By the final state, the performance benefit is about 2.7 (or 500-fold) for Investigator and about 3.0 (or 1000-fold) for Tender. This is a result that favors multiaspect classification but relies on the assumption (required of the product rule) that measurements from different aspects are independent, which may not be necessarily true since radar measurements of a target from different aspects are clearly correlated to some extent. This is the problem that we will attempt to address next. A summary of the multiaspect SVM procedure is shown in Algorithm 1.
**Algorithm 1:** Multiaspect SVM**Input:** Preprocessed radar training data**Output:** Log-likelihood ratio (LLR) for each target **TRAINING**:**for** i:M aspects **do**Perform training of SVM: ***w****_i_, b_i_*Compute target probabilities, *P_i_(X|O)* from PPV and NPV statisticsCarry out Laplace smoothing: ${\hat{\theta }}_{i}$**end for**Sort aspects from highest value to lowest value **TESTING**:Determine aspects required for data collection: *N***for** i:N aspects **do****for** j:P testPoints **do**Make radar measurementPreprocess data: ***x***Perform classification: *h(**x**)*Lookup *P_j_*(*X*|*O*) from training data for each targetApply sum rule to determine average *P_i_(X|O)* for each target**end for**Apply product rule to determine aggregate: *P(X|O)* for each targetUpdate LLR**end for**

### 4.4. Combined Feature Space SVM

The previous section implemented separate SVM classifiers at each target aspect and combined results using the sum and product rules. However, this approach assumed independent measurements, which may not necessarily be the case. It is therefore now desirable to find a more appropriate means of combining results from different aspects that is effective and can provide a more accurate confidence measure of performance improvements.

One such solution is to combine feature spaces from the most valuable aspects and perform a single classification. This would allow the SVM to inherently perform the combination of aspects, and performance improvements could be accurately obtained from the classifier’s performance statistics. As an additional benefit, such an approach would reduce the prediction error when compared to the sum and product rules [43].

Prior to combining feature spaces, it is desirable to identify the most valuable features so that the resulting feature space is not excessively large and contains only the important features. There are many options for performing feature selection for SVM classification such as F-score and random forests [44], though we have used the Kolmogorov–Smirnov (K–S) test; this is a nonparametric approach that is consistent with the data-driven methodology employed in this paper. Since it relies on quantifying differences in the cumulative distribution functions of features from the two targets, it is intuitively comparable to the workings of a linear SVM in high-dimensional feature space.

The Kolmogorov–Smirnov test is defined here as:

(18) $$F_{n}\left(x\right)=\frac{1}{n}\overset{n}{\underset{i=1}{\sum }}I_{\left[-\infty ,x\right]}\left(X_{i}\right)$$

where $F_{n}\left(x\right)$ is the empirical distribution function, $I_{\left[-\infty ,x\right]}\left(X_{i}\right)$ is the indicator function such that $I_{\left[-\infty ,x\right]}=1$ for $X_{i}\leq x$ and $I_{\left[-\infty ,x\right]}=0$ for $X_{i}>x$.

The Kolmogorov–Smirnov statistic is then:

(19) $$D_{n,m}=\underset{x}{sup}\left|F_{1,n}\left(x\right)-F_{2,n}\left(x\right)\right|$$

where *sup* is the supremum function.

**Figure 13:**
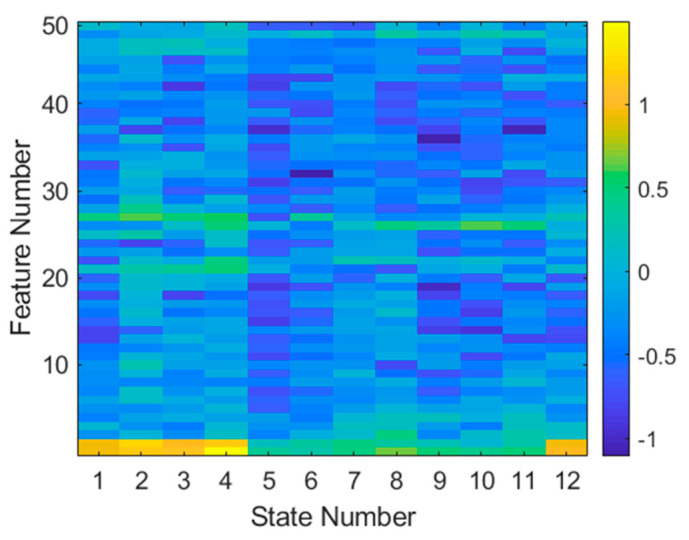
Log Kolmogorov–Smirnov statistic for every feature and aspect. The larger (yellow) values indicate features that differ the most between targets.

The logarithmic K–S statistic generated from all data of both targets is shown in Figure 13. The highest value feature (the one that differs the most between targets) according to the K–S statistic is feature 1 of state 4. The second most valuable feature is feature 2 from state 2. It is evident that high-importance features (in yellow) are at the bottom and middle of the feature space (which is in the frequency domain), indicating that target magnitude (radar cross-section) plays an important role in this classification.

**Figure 14:**
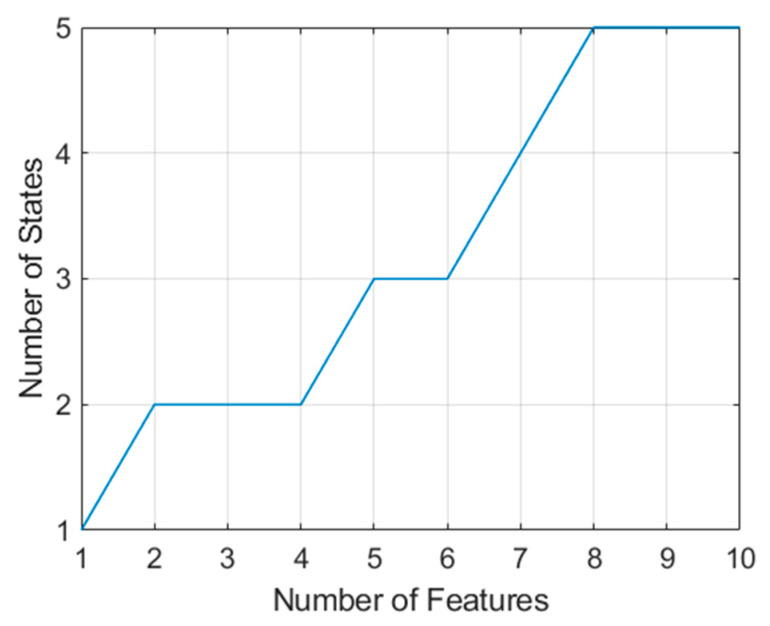
Growth of states required to obtain (measure) the highest value features according to the log Kolmogorov–Smirnov statistic.

These features have been ranked in order from most valuable to least valuable. Figure 14 indicates the number of states that contain the top 10 features. It is evident that in order to collect the best 10 features, features from a total of five aspects must be combined (if the radar is limited to fewer aspects than this, there is no reason why other high-value features from the same aspects could not be combined).

Since we are combining features from different aspects (corresponding to different training and test sets), and there is nothing that links the nth sample from one test set with the nth sample of another (since the measurements for different aspects were carried out at different times), we randomly selected which training and test points were combined between datasets. This allowed new, much larger datasets to be generated. In this case, we used training and test sets of 1000 samples (i.e., feature vectors) for each of the 10 simulations shown. The SVM classification was then performed on a reduced and concatenated feature vector:

(20) $$x_{K-S}\left(a\right)\in \{x\{sort_{a}(D_{n,m})\}\}$$

where $x_{K-S}\left(a\right)$ is the *K* − *S* indexed feature vector of length *a* from the original preprocessed samples x, and $sort_{a}()$ represents the indices corresponding to the largest *a* values of $D_{m,n}$ from all sectors, ∀1<m<12.

**Figure 15:**
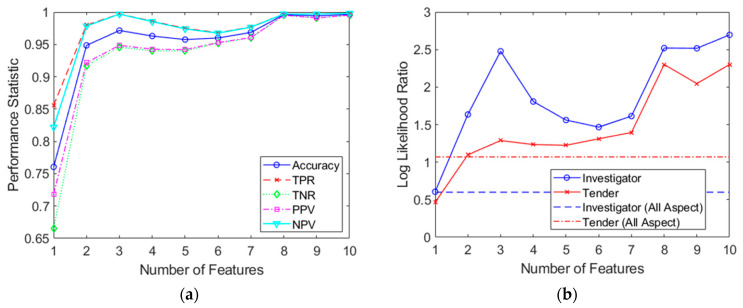
Combined feature space classifier results shown for feature spaces of 1 to 10 in length. (**a**) Resulting performance statistics; (**b**) calculated log-likelihood ratios for both targets.

Figure 15 shows the results from classifications performed on combined features spaces, of length *a* = 1 to 10. Remarkably, Figure 15a shows that for feature spaces with lengths of only 2, an accuracy of about 0.95 can be achieved. As the feature vector grows to about 10 in length, the performance stabilizes and approaches perfect classification. Figure 15b shows the corresponding log-likelihood ratio, as previously depicted in Figure 12b. Once again, performance from the simple, full dataset classification has been incorporated using dashed lines. As before, it is evident that the multiaspect approach is of benefit, as is evident in Figure 15b; only two features (across two aspects) were required to match the performance of the conventional full dataset classifier. However, the maximum log-likelihood ratio possible was significantly less so than using the sum and product rules in the previous section This may indeed highlight that the measurements across aspects are dependent on each other. By the tenth feature, the log-likelihood improvement was approximately 2.1 (or a 126-fold improvement over conventional full dataset classification) for Investigator and 1.2 (or a 16-fold improvement over conventional full dataset classification) for Tender. However, while the independence issue was overcome, the caveat now is that since measurements of different aspects have been carried out at different times (there was only one radar system available), there is no guarantee that the internal state of the target and environment is the same (for example, if the sea-state changed or a door was opened on the ship in between measurements). This new assumption, that data are generated from a stationary process, replaced the previous assumption of independence. Nonetheless, these results probably offer a more realistic insight into the benefits that can be achieved from multiaspect classification in the context of this paper. A summary of the combined feature space procedure is shown in Algorithm 2.
**Algorithm 2:** Combined Feature Space SVM**Input:** Preprocessed radar data**Output:** Log-likelihood ratio (LLR) for each target **TRAINING:****for** i:M aspects **do**Calculate distribution function for each target: *F_n_(x)*Calculate K–S statistic: *D_n,m_***end for**Determine number of features to useConcatenate features into feature vector: ***x***Randomly generate training set and validation setPerform training of SVM: ***w****_i_, b_i_*Sort aspects from highest value to lowest valueDetermine LLR for each target based on validation set **TESTING:**Determine aspects required for data collection: *N***for** i:N aspects **do****for** j:P testPoints **do**Make radar measurementPreprocess data: ***x*****end for****end for**Perform classification: *h(**x**)*Lookup LLR for each target

## 5. Discussion

Millimeter-wave measurements of two controlled maritime targets were conducted at W-band a short distance (less than one kilometer) off the coast, with the radar mounted on top of a control tower. These measurements were tailored to collect data required for this research and included full target attitude and heading. The data consisted of HRRPs and were preprocessed by performing range alignment and taking FFTs, with all classifications carried out in the frequency domain. While this data were sufficient for classification, the datasets later proved to be highly imbalanced (between aspects) and too small for an ideal investigation of classification approaches. However, these shortcomings turned out to be fortuitous in that they focused and steered research towards particular solutions.

Three methods based on the linear SVM classifier were contrasted. The first (reference) method was a simple linear SVM classification of all test data, which yielded an accuracy of 0.87. This approach approximately matched the performance of a basic power level classification conducted early in the paper, which yielded an accuracy of 0.86. This method did not allow for selection of aspects since there was effectively only one sector of concern.

The second SVM method split data into 12 (30°) sectors and implemented an SVM classifier at each sector. Using the PPV and NPV statistics, target probabilities could be easily obtained, which allowed the most valuable aspects to be identified. This method was not well-suited to small datasets and smoothing had to be carried out to prevent zero and one target probabilities. However, the approach still achieved excellent performance. By prioritizing aspects and using the sum rule to combine target probabilities within an aspect, and the product rule to combine target probabilities from different aspects, monotonically increasing target probabilities were achieved for the three highest priority sectors. When continuing to accumulate target probabilities from all other aspects, a 500-fold improvement for one target and 1000-fold improvement for the other target could be achieved when compared to the reference SVM classifier. However, this result was questionable since the product rule assumed independence between aspects which could not be guaranteed.

In order to address the independence concerns, a third method was developed that combined feature spaces from several aspects, with the SVM performing the multiaspect combination of probabilities inherently. As part of this process, the Kolmogorov–Smirnov test was applied to prioritize features and reduce dimensionality. This approach also allowed features from different aspects to be randomly matched, increasing the size of the effective dataset by many orders of magnitude. Consequently, this approach is well-suited to small and imbalanced datasets. Excellent performance was achieved using between two and ten features, making the approach suitable for resource-limited implementations while maintaining generality. A 126-fold improvement was demonstrated for one target, and a 16-fold improvement for the other target when compared to conventional, full dataset classification. A summary of relative performance for all three SVM approaches is shown in Table 7.

In terms of implementation, there were slight differences evident between approaches. The conventional full dataset approach is straightforward but requires retraining with any addition of new data. The advantage of this approach is that although performance is not high, there are no assumptions made of independence so the likelihood of the classifier performing correctly is known in advance.

The multiaspect approach is able to predict the value of taking measurements at each aspect. Consequently, fewer measurements are required to achieve the same level of performance as conventional classification. The addition of new data only requires retraining at the aspects where the data were collected. However, this method was not well-suited to small and imbalanced datasets where smoothing had to be carried out to improve reliability. Furthermore, target confidence levels assume independence between aspects and may be misleading.

The combined feature space approach also allows for the identification of the most valuable aspects in advance. Furthermore, it is well-suited to small and imbalanced datasets. The dimensionality reduction makes it suitable for large feature spaces, though this adds to the complexity of the approach. The approach is particularly well-suited to use cases where radar transmissions must be minimized (only two transmissions were required for accurate classification). Confidence in classifier output is likely to be in between the other two methods since the assumption of a stationary process may partially hold in practice. New data will generally require full retraining.

Current state of the art approaches for implementing HRRP radar classification generally involve deep belief networks or autoencoders. Both approaches suffer from training complexity and the need for large datasets. Furthermore, currently they do not appear to offer significant improvements in classification performance for tasks of comparable complexity. However, these techniques will be helpful in overcoming some of the future challenges of multiaspect radar classification; as use cases evolve to include complex libraries of targets, distributed sensors and diverse environments, the generalization properties of neural networks are bound to play an important role in replacing bespoke preprocessing. As research continues and these techniques and tools mature, they will offer more robust performance while largely removing the need for specialized engineering of the classifier.

Further work will include gathering data from more targets and applying some of the numerous neural network approaches available. Many of these techniques have not been investigated in the context of distributed multiaspect scenarios.

## 6. Conclusions

This paper utilized radar measurements at W-band to demonstrate two multiaspect target classification approaches based on the SVM classifier. The first approach relied on training at individual aspects, combining target probabilities and ranking aspects in terms of their accuracy. The second approach relied on concatenating features across aspects to combine target probabilities. Compared to conventional full dataset classification, these approaches improved accuracy by 6% on average when utilizing only two 30° sectors. Additionally, these approaches offer many other benefits such as the ability to prioritize aspects, reduce transmissions, reduce training requirements, and fuse data from multiple aspects or sensors. As new deep learning techniques mature, this work will be extended to reduce the need for specialized preprocessing and cater for more complex scenarios to enable future UAS implementations.

## Figures and Tables

**Table 1:** Radar and attitude and heading reference systems (AHRS) data collection parameters.

Parameter	Data or Method Used
AHRS Data Collected	Yaw/heading, pitch, roll, position
AHRS Collection Interval	17.24 ms
Radar Data Collection	HRRP
Centre Frequency	94 GHz
Waveform Type	FMCW sawtooth modulation, 20 ms upward chirp
Waveform Bandwidth	400 MHz
Peak Power	200 mW
Antenna Gain	44 dBi transmit, 44 dBi receive
Waveform Frame	16 pulses end-to-end (320 ms)
Radar Collection (Frame) Interval	1.48 s

**Table 2:** Confusion matrix and performance derivations for the mean power level hard threshold classifier using a 7.7 dB threshold.

		**Predicted**	
		**Investigator**	**Tender**
**Actual**	**Investigator**	459	57
	**Tender**	87	439
**Accuracy**		0.86	
**True Positive Rate (Investigator)**		0.89	
**True Negative Rate (Tender)**		0.83	
**Informedness**		0.72	

**Table 3:** Confusion matrix and performance derivations corresponding to log classification with normalization.

		**Predicted**	
		**Investigator**	**Tender**
**Actual**	**Investigator**	28	5
	**Tender**	2	17
**Accuracy**		0.87	
**True Positive Rate (Investigator)**		0.85	
**True Negative Rate (Tender)**		0.89	
**Informedness**		0.74	

**Table 4:** Confusion matrix and performance derivations corresponding to log classification without normalization.

		**Predicted**	
		**Investigator**	**Tender**
**Actual**	**Investigator**	23	6
	**Tender**	1	22
**Accuracy**		0.87	
**True Positive Rate (Investigator)**		0.79	
**True Negative Rate (Tender)**		0.96	
**Informedness**		0.75	

**Table 5:** Confusion matrix and performance derivations corresponding to linear classification with normalization.

		**Predicted**	
		**Investigator**	**Tender**
**Actual**	**Investigator**	16	13
	**Tender**	4	19
**Accuracy**		0.67	
**True Positive Rate (Investigator)**		0.55	
**True Negative Rate (Tender)**		0.83	
**Informedness**		0.38	

**Table 6:** Confusion matrix and performance derivations corresponding to linear classification without normalization.

		**Predicted**	
		**Investigator**	**Tender**
**Actual**	**Investigator**	18	3
	**Tender**	3	28
**Accuracy**		0.88	
**True Positive Rate (Investigator)**		0.86	
**True Negative Rate (Tender)**		0.90	
**Informedness**		0.76	

**Table 7:** Summary of performance and general characteristics for all three SVM classification approaches tested.

	Full Dataset			Multiaspect		Combined Feature Space	
	Lin.	RBF	Poly.	First Aspect	First Two Aspects	First Aspect	First Two Aspects
**Accuracy**	0.87	0.86	0.84	0.95	0.90	0.76	0.95
**Final LLR (Linear)**	1.2 (16)			3.5 (3162)		2.5 (316)	

## Data Availability

The data presented in this study are available on request from the corresponding author. The data are not publicly available due to confidentiality.
